# Liquid Microbial-Enzymatic Co-Fermentation of Walnut and Sesame Meals and Its Effects on Nutrient Digestibility in Growing Pigs

**DOI:** 10.3390/ani16020220

**Published:** 2026-01-12

**Authors:** Caimei Wu, Meihong Li, Ziyun Zhou, Kun Zhang, Yixuan Zhou, Fali Wu, Jie Yu, Jian Li, Ruinan Zhang, Hua Li, Jiayong Tang, Lianqiang Che, Yang Lyu

**Affiliations:** Key Laboratory for Animal Disease-Resistance Nutrition and Feedstuffs of China Ministry of Agriculture and Rural Affairs, Institute of Animal Nutrition, Sichuan Agricultural University, Chengdu 611130, China

**Keywords:** unconventional feed, liquid fermentation, nutritive value, ileal digestibility, growing pigs

## Abstract

This study first adopted a liquid microbial-enzymatic co-fermentation process to enhance the nutritional value of walnut meal (WM) and sesame meal (SM), and systematically evaluated its effect on the nutrient digestibility of growing pigs. WM and SM are two underutilized high-protein by-products, whose application is hindered by anti-nutritional tannin and fiber. Optimal fermentation parameters were determined via single-factor experiments and response surface methodology, utilizing a consortium of *Lactobacillus* I, *Candida utilis*, and protease. Fermentation significantly reduced tannin (39.41% in WM) and crude fibre (28.79% in WM), reduced tannin (18.67% in SM) and crude fibre (4.00% in SM), while elevating crude protein (10.63% in WM, 7.47% in SM) and acid-soluble protein in both WM and SM. Results of the microstructure of fermented WM and SM revealed structural loosening, surface porosity, and polysaccharide degradation. Microbial community shifts highlighted the dominance of *Lactobacillus* and *Bacillus* in fermented substrates. In growing pigs, fermented WM and SM exhibited improved standardized ileal digestibility (SID) of key amino acids (threonine, tryptophan, valine; *p* < 0.05), alongside enhanced digestible energy (DE) and metabolizable energy (ME) for SM (*p* < 0.05). These findings demonstrate that liquid co-fermentation effectively degrades anti-nutritional factors, enhances nutrient bio-availability, and positions WM and SM as viable alternatives to conventional protein sources in swine diets, supporting strategies to reduce reliance on soybean meal.

## Simple Summary

This research developed a liquid microbial-enzyme co-fermentation process to enhance the nutritional value of walnut meal and sesame meal. The process significantly reduced antinutritional factors such as tannins and crude fiber, while increasing crude protein and acid-soluble protein content. In trials with growing pigs, the fermented products improved the digestibility of key amino acids and enhanced dietary energy utilization. The findings indicate that fermented walnut meal and sesame meal can serve as viable protein alternatives to soybean meal in pig diets, and provide technical support for expanding growing pig feed protein sources and promoting the rational and efficient application of these two meals in growing pig feed.

## 1. Introduction

Walnut meal (WM) and sesame meal (SM), by-products of walnut oil and sesame oil extractions, are produced in substantial quantities in China and represent a potentially valuable high-protein feed resource for livestock and poultry [1,2]. WM contains roughly 40% crude protein and a balanced amino acid profile, and the protein content of SM can reach 40~50%. Both are higher than those of soybean meal, cottonseed meal, and rapeseed meal [1,2]. Studies suggest that supplementing diets with flaxseed and WM can effectively reduce nitrogen excretion, enhance nitrogen utilization efficiency, and improve average daily gain (ADG) in piglets [3]. Liu et al. (2024) [4] reported that incorporating 5% walnut kernel cake into pig diets significantly increased crude fat content and back fat thickness, indicating that WM promotes fat deposition. Pérez-Trejo et al. (2022) [2,5] observed that fattening lambs fed a basal diet supplemented with sesame meal instead of soybean meal had the highest gross profit margin. Farrokhi et al. (2021) [6] found that feeding broiler chickens with a diet supplemented with sesame meal significantly increased their daily feed intake.

However, the utilization of WM and SM in animal feed is limited by their high tannin (derived from coats) and fibre content [7]. Tannins, characterized by their astringent taste and strong affinity for proteins and polypeptides, readily form complexes that reduce protein digestibility in animals [8]. To enhance the nutritional value of WM and SM, feedstuff fermentation has emerged as a promising approach [8]. Liquid-state fermentation (LFF), involving the mixing of feed with water at a ratio of 1:1.5 to 1:4.0, can be conducted spontaneously or through inoculation with specific microorganisms [9]. During LFF, microbial enzymes degrade toxins and anti-nutritional factors in the feed, while generating beneficial metabolites such as volatile fatty acids, vitamins, and bacteriocins [10]. Compared to solid-state fermentation, LFF offers superior control over the fermentation environment and enhances palatability, leading to increased feed intake [11]. Previous studies have demonstrated that LFF significantly reduces *Enterobacteriaceae* abundance while increasing *Lactobacillus* abundance in the pig gut [12]. Notably, this modulation of gut microbiota can be transmitted from gestating sows to their offspring, reducing piglet diarrhoea incidence [13]. Furthermore, LFF has been shown to significantly improve feed intake, nutrient digestibility, and ADG in growing–finishing pigs [14].

Enzymatic hydrolysis involves the addition of specific enzymes to the feed to achieve targeted effects [15]. For example, phytase degrades phytic acid, improving phosphorus availability, while proteases break down large protein molecules into readily absorbable peptides [16]. Fermentation, in contrast, utilizes enzyme-active substances produced through microbial metabolism to degrade anti-nutritional factors and enhance nutrient content [17]. Furthermore, fermented feed can positively influence gut microbiota, offering a potential alternative to antibiotics [18]. However, relying solely on enzyme preparations to improve feed materials can be costly, while microbial fermentation alone may lead to excessive nutrient depletion [19]. Co-fermentation technology, which synergistically combines the benefits of both enzymes and microorganisms, represents a promising approach for efficient and cost-effective feed pre-treatment [20]. Specifically, lactic acid bacteria (LAB) produce substantial amounts of lactic acid, rapidly decreasing the fermentation pH and inhibiting the growth of undesirable bacteria [21]. Moreover, research indicates that yeast can significantly increase the crude protein content of feed; consequently, yeast and LAB are frequently employed in LFF [22]. Notably, *Saccharomyces cerevisiae* (brewer’s yeast) and *Lactobacillus plantarum* also exhibit the capacity to degrade tannin through the production of metabolites with tannase activity [23,24].

Despite its potential as a significant unconventional protein feed resource in China, the utilization of WM and SM is limited by their high tannin and fibre content [7]. Effective methods to mitigate these anti-nutritional factors and enhance the quality and nutritional value of both remain limited. Therefore, it is of great significance to explore the fermentation optimization effects and nutritional value of WM and SM, so as to enrich the diversity of protein sources in swine production. We hypothesize that liquid-state microbial-enzymatic co-fermentation can improve the nutrient digestibility of WM and SM by degrading anti-nutritional factors and modifying the substrate structure. In general, this study aimed to develop a liquid-state microbial-enzymatic co-fermentation process to improve the nutritional profile of WM and SM. Specifically, we sought to evaluate the impact of this co-fermentation process on nutrient digestibility, DE, and ME in growing pigs. The results will provide a scientific foundation for the enhanced and efficient utilization of WM in swine diets and contribute to the enrichment of the diversity of protein feed resources.

## 2. Materials and Methods

### 2.1. Screening of Fermentation Strains

#### 2.1.1. Activation and Scale-Up Cultivation of Strains

*Lactobacillus* I (CGMCC 1.12934), *Lactobacillus* II (CGMCC 1.557), *S. cerevisiae* (Bio-64452), and *C. utilis* (BNCC-336517) were obtained from our previous studies. YPD (Yeast Extract Peptone Dextrose Medium) and MRS (de Man, Rogosa and Sharpe Medium) were purchased from Hope Bio-Technology (Co., Ltd., Qingdao, China). To prepare the cultures, frozen stocks stored at −80 °C were streaked onto agar plates (using 20 μL inoculum per strain) and incubated inverted under appropriate conditions for colony formation. Single colonies of each strain were then transferred to 20 mL of either YPD (for *S. cerevisiae* and *C. utilis*) or MRS (for *Lactobacillus* I and II) liquid broth. *S. cerevisiae* and *C. utilis* were cultivated at 30 °C and 200 rpm, while *Lactobacillus* I and II were cultivated at 37 °C and 200 rpm. After 12 h of cultivation, 1 mL aliquots of each culture were used to inoculate 100 mL of fresh YPD or MRS broth in shake flasks, and scale-up cultivation was performed under the same temperature and agitation conditions as the initial activation process.

#### 2.1.2. Correlation Between OD600 and Strain Concentration

During cultivation, OD600 values were monitored by spectrophotometry; when readings reached 0.5–1.5, 1 mL aliquots were withdrawn from each culture flask and serially diluted to 10^4^, 10^5^, and 10^6^. Subsequently, 100 μL aliquots from each dilution were transferred to agar plates, spread using sterile glass beads, and incubated for 24 h prior to colony counting. The cell concentration (CFU/mL) was calculated as follows: (Colony count × 10 × Dilution factor), with each sample analyzed in triplicate. Cultivation time, OD600 values, and colony counts were recorded. Regression analysis was performed using OD600 values as the independent variable (*x*-axis) and cell concentrations as the dependent variable (*y*-axis) to establish a correlative regression equation.

#### 2.1.3. Screening of Fermentation Strains

Finely ground walnut and sesame meal (20 g) was weighed into conical flasks, adjusted to a solid-to-liquid ratio of 1:4 (wt/vol), and inoculated with 2 × 10^9^ CFU/g of each of the four pre-cultured microbial suspensions, with three replicates per group. Yeast fermentation groups (*S. cerevisiae* and *C. utilis*) were incubated at 30 °C for 48 h, while *Lactobacillus* fermentation groups (*Lactobacillus* strains I and II) were incubated at 37 °C for 48 h. After fermentation, the material was oven-dried at 65 °C to an air-dry basis, ground, and sieved through a 40-mesh screen. Tannin content was measured (triplicate measurements per group), and the strain demonstrating the most significant anti-nutritional factor degradation was selected for subsequent experimentation.

Using the optimal medium formulation identified via preliminary single-factor experiments, the influence of key parameters (strain ratio, inoculum size, enzyme supplementation level, solid-to-liquid ratio, fermentation temperature, and duration) on acid-soluble protein content in fermented WM and SM (FWM and FSM) was evaluated employing a one-variable-at-a-time approach; factor levels are detailed in Supplementary Table S1.

Optimization of fermentation parameters (inoculum size, solid-to-liquid ratio, temperature, and time) was subsequently performed using a four-factor, three-level Box–Behnken design with acid-soluble protein content as the response variable, including five replicates at the central point; the experimental design is presented in Supplementary Table S2.

All samples were ground to pass through a 0.3 mm sieve and analyzed for dry matter (DM; AOAC method 930.15), crude protein (CP; method 990.03), ash (method 942.15), and ether extract (EE; method 920.39) according to standard methods of the Association of Official Analytical Chemists. Crude fibre (CF), neutral detergent fibre (NDF), and acid detergent fibre (ADF) values were determined using a fibre analyser. Amino acid composition was analyzed as follows: after hydrolysis with 6 mol/L HCl at 110 °C for 24 h, 15 amino acids (excluding methionine and cysteine) were quantified using an automatic amino acid analyzer; tryptophan was determined via high-performance liquid chromatography following hydrolysis with 0.1 mol/L lithium hydroxide at 110 °C for 20 h; methionine and cysteine were analyzed using the automatic amino acid analyzer (L-8900, Hitachi High Tech, Tokyo, Japan) after performic acid oxidation for 18 h and subsequent hydrolysis in 7.5 mol/L hydrochloric acid at 110 °C for 24 h.

For scanning electron microscopy, samples were affixed to stubs using conductive adhesive tape, dispersed uniformly onto the tape, and gently purged with compressed air to remove loose particles prior to imaging.

For the Fourier transform infrared spectrophotometer (FTIR, Nicolet iS10, Thermo Fisher Scientific Inc., Waltham, MA, USA), samples dried at 65 °C and ground through a 60-mesh sieve were meticulously mixed with spectroscopic-grade potassium bromide (KBr; 2 mg sample to 200 mg KBr) under an infrared lamp to maintain dryness, ensuring complete encapsulation by KBr; the mixture was then pressed into translucent, homogeneous pellets under controlled conditions. FTIR spectra were acquired in the range of 4000–400 cm^−1^ at 4 cm^−1^ resolution, accumulating 32 scans per spectrum using an FTIR spectrometer. The resulting spectra were deconvoluted using OMNIC software version 7.1 and visualized in Origin software version 10.1 to generate the FTIR profiles.

The microbial community of fermented vs. unfermented samples was analyzed by 16S rRNA gene sequencing. Total bacterial DNA was extracted using a commercial kit (Majorbio Technology Co., Ltd., Shanghai, China). DNA size and integrity were assessed by electrophoresis on 1% agarose gels stained with ethidium bromide. DNA concentration and purity were determined by spectrophotometric measurement at 234 nm, 260 nm, and 280 nm. Subsequently, DNA samples were sent to Majorbio Technology for library preparation, sequencing (Illumina MiSeq, San Diego, CA, USA), and bioinformatic analysis (QIIME 2).

### 2.2. Amino Acids Digestibility in the Ileum of Growing Pigs

#### 2.2.1. Experimental Design

The experiment was performed following the Chinese Guidelines for Animal Welfare set by the National Institute of Animal Health and approved by the Animal Care and Use Committee of Sichuan Agricultural University (Approval No.20240155). Ten castrated male Duroc × Landrace × Yorkshire crossbred pigs with an initial body weight of 22.46 ± 1.13 kg were surgically fitted with terminal ileal T-cannulas, following the procedure described by Stein [25]. The cannulated pigs were then randomly assigned to two groups (fermented diet vs. unfermented diet) and incorporated into a 5 × 5 Latin square design that also included two other feed ingredients. This design resulted in five experimental periods, each lasting 8 days and comprising a 3-day adaptation period, a 3-day pre-collection period, and a 2-day ileal digesta collection period. Within each period, each group received one nitrogen-free diet and four test diets, two of which contained WM and SM under investigation. The detailed formulations for the WM and SM diets, fermented WM and SM diets, and the nitrogen-free diet are presented in Supplementary Table S3.

#### 2.2.2. Sample Collection

Ileal digesta collection began 2 h after feeding each day. Collection bags were attached to the exterior of the ileal cannula using rubber bands and replaced every 20 to 30 min. All collected samples were immediately labelled and stored at −20 °C. Following the trial, digesta samples from each animal, period, and diet were pooled, freeze-dried under vacuum, and ground to pass through a 40-mesh sieve. The resulting powder was then stored at −20 °C until analysis. Test diet samples, collected periodically throughout the trial, underwent a parallel process: freeze-drying, grinding to pass through a 40-mesh sieve, and storage at −20 °C for subsequent analysis.

#### 2.2.3. Analysis and Calculations

Experimental diets and ileal digesta samples were ground to pass through a 1 mm sieve prior to analysis. The content of CP and eighteen amino acids (AAs) was then determined. Chromic oxide (Cr_2_O_3_) content in both feed and ileal digesta was quantified using the spectrophotometric method, following the protocol outlined in GB/T 5009.246-2016 [26]. These values were subsequently used to calculate:

(1) Apparent ileal digestibility (AID%) of amino acids = [1 − (AA content in ileal digesta/AA content in test diet) × (Cr_2_O_3_ content in ileal digesta/Cr_2_O_3_ content in test diet)] × 100.
(2) Basal endogenous ileal amino acid losses (BIAA) = AA content in ileal digesta when feeding the nitrogen-free diet × (Cr_2_O_3_ content in the nitrogen-free diet/Cr_2_O_3_ content in ileal digesta when feeding the nitrogen-free diet).
(3) Standardized ileal digestibility (SID%) of amino acids = AID + (BIAA/AA content in test diet) × 100.

### 2.3. Nutrient Digestibility, Digestible Energy, and Metabolic Energy

#### 2.3.1. Experimental Design

In a separate experiment conducted concurrently with trials involving other feed ingredients, ten healthy castrated male Duroc × Landrace × Yorkshire crossbred pigs (initial body weight: 15.34 ± 0.55 kg) were randomly assigned to two groups within a replicated 5 × 5 Latin square design. Each group received five experimental diets: a basal diet and four test diets, including the WM and SM diets. The experiment consisted of five periods, each comprising a 3-day adaptation phase, a 3-day pre-collection phase, and a 2-day collection phase, during which faeces and urine were collected and stored at −20 °C for subsequent analysis. Pigs were housed individually in metabolic cages (2.5 × 1.8 × 0.8 m) with ad libitum access to water and a restricted daily feed allowance of 4% of body weight, divided into two equal meals provided at 08:00 and 14:00. Metabolic cages were alternately sanitized using at least two types of disinfectant every other day, and ambient temperature was maintained at approximately 26 °C. The detailed formulations for the basal diet and walnut meal diets are presented in Supplementary Table S4. All diets were formulated on an air-dry basis, and the inclusion rates of vitamin and mineral premixes met or exceeded the nutrient requirements for 11–25 kg growing pigs.

#### 2.3.2. Sample Collection

Experimental diets for each period were thoroughly mixed, freeze-dried, and ground to pass through a 40-mesh sieve. The resulting powder was then labelled and stored at −20 °C until analysis. Fecal collection began 2 h after feeding, with samples stored at −20 °C. At the conclusion of the trial, fecal samples from each period were pooled, freeze-dried to a constant weight, and equilibrated for 24 h to determine initial moisture content. These dried samples were then ground to pass through a 40-mesh sieve, labelled, and stored at −20 °C. For urine collection, six layers of gauze were secured to the funnel of each metabolic cage, positioned above polypropylene containers pre-charged with 50 mL of 10% sulfuric acid. Urine was collected daily at consistent times, and the total volume was recorded using a graduated cylinder. Following a secondary filtration through six gauze layers, aliquots were labelled and stored at −20 °C. At the conclusion of the trial, all urine samples were pooled by animal, homogenized, and subjected to a tertiary filtration through eight gauze layers. The resulting filtrate was then aliquoted into equal volumes in 50 mL centrifuge tubes and frozen for subsequent analysis.

#### 2.3.3. Analysis and Calculations

All diets and fecal samples were analyzed for moisture, CP, EE, CF, crude ash, calcium, phosphorus, and gross energy, following the procedures as previously detailed. Urinary energy was determined by saturating 0.25 g circular filter paper discs (pre-folded into a tricorn shape within iron crucibles) with incremental aliquots of urine, totalling 10–15 mL. After each addition, samples were dried at 65 °C. The saturated paper discs were then subjected to gross energy analysis using bomb calorimetry, with multiple blank determinations performed for calibration. The content of Cr_2_O_3_ was quantified using the colorimetric method specified in GB/T 5009.246-2016 [26]. The following equations were used for calculations:

(1) Nutrient digestibility (%) = [(Nutrient intake − Faecal nutrient output)/Nutrient intake] × 100.
(2) Apparent digestible energy (DE, MJ/kg) = (Gross energy intake − Faecal energy)/Total diet intake.
(3) Apparent metabolizable energy (ME, MJ/kg) = (Gross energy intake − Faecal energy − Urinary energy)/Total diet intake.
(4) Test ingredient nutrient digestibility (%) = [100 × (Test diet nutrient digestibility − Basal diet nutrient digestibility)/(Proportion of nutrient contributed by test ingredient in test diet)] + Basal diet nutrient digestibility.
(5) Test ingredient DE or ME (MJ/kg) = [(Test diet DE or ME value − Basal diet DE or ME value × A)/B], where A represents the proportion of basal diet in the test diet and B represents the proportion of the test ingredient in the test diet (A + B = 100%).

### 2.4. Statistical Analysis

The normality of residuals was confirmed using Shapiro–Wilk tests (all *p* > 0.05), and the homogeneity of variances was verified using Levene’s test (*p* > 0.05). Regression model construction and analysis of variance (ANOVA) for the response surface methodology (RSM) experiments were performed using Design-Expert software (Version 12.0.0). Statistical analysis of the experimental results was conducted using the General Linear Model procedure in SPSS Statistics software (Version 24.0). Statistical significance was declared at *p* < 0.05, and Turkey’s Honest Significant Differences test was used for post hoc comparisons. Data are presented as mean ± standard error of the mean (SEM). A *p*-value < 0.05 was considered statistically significant, while 0.05 ≤ *p* < 0.10 indicated a trend towards significance.

## 3. Results

### 3.1. Results of WM and SM Fermentation

#### 3.1.1. Screening of Fermentation Strains

**Figure 1:**
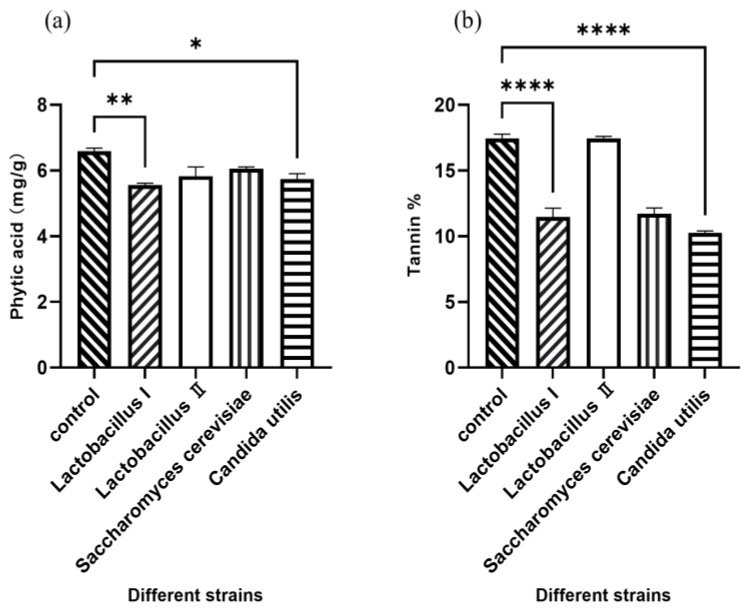
The levels of phytic acid (**a**) and tannin (**b**) in WM and SM after fermentation. * *p* < 0.05, ** *p* < 0.01, **** *p* < 0.001.

Polynomial equations describing the correlation between OD values and viable cell concentration for activated and amplified cultures of each strain are presented in Table 1. Figure 1 demonstrates the effect of inoculating four different microbial strains on the concentration of phytic acid and tannin in fermented WM and SM. Compared to the control group, *Lactobacillus* I and *C. utilis* significantly decreased the concentration of phytic acid and tannin (*p* < 0.05), indicating that 2 × 10^9^ CFU/g of *Lactobacillus* I and *C. utilis* were optimal.

#### 3.1.2. Fermentation Single Factor Optimization

**Figure 2:**
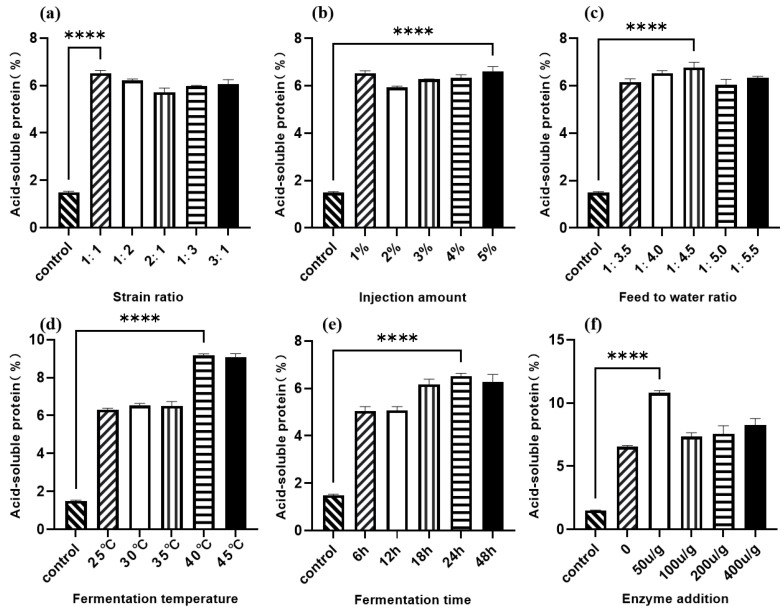
Single-factor optimization of walnut meal fermentation with acid-soluble protein content as the dependent variable. (**a**) Strain ratio; (**b**) injection amount; (**c**) feed to water ratio; (**d**) fermentation temperature; (**e**) fermentation time; (**f**) enzyme addition. **** *p* < 0.001.

As shown in Figure 2a, the 1:1 ratio of *Lactobacillus* I to *C. utilis* resulted in the greatest increase in acid-soluble protein (ASP) content compared to the control. Figure 2b shows that all five tested inoculum volumes significantly increased ASP levels compared to the control, with a 5% (*v*/*w*) inoculum concentration yielding the highest ASP. The material-to-water ratio’s effect on walnut meal fermentation is presented in Figure 2c, with a 1:4.5 ratio resulting in maximum ASP content. As illustrated in Figure 2d, fermentation temperatures between 25 °C and 35 °C maintained ASP content around 6%, while higher temperatures (40 °C and 45 °C) significantly increased ASP production, with 40 °C exhibiting the greatest effect. Figure 2e demonstrates that ASP content increased progressively with fermentation time, reaching optimal levels at 24 h. Finally, Figure 2f shows that protease supplementation at 50 U/g produced the greatest increase in ASP content among the tested concentrations.

**Figure 3:**
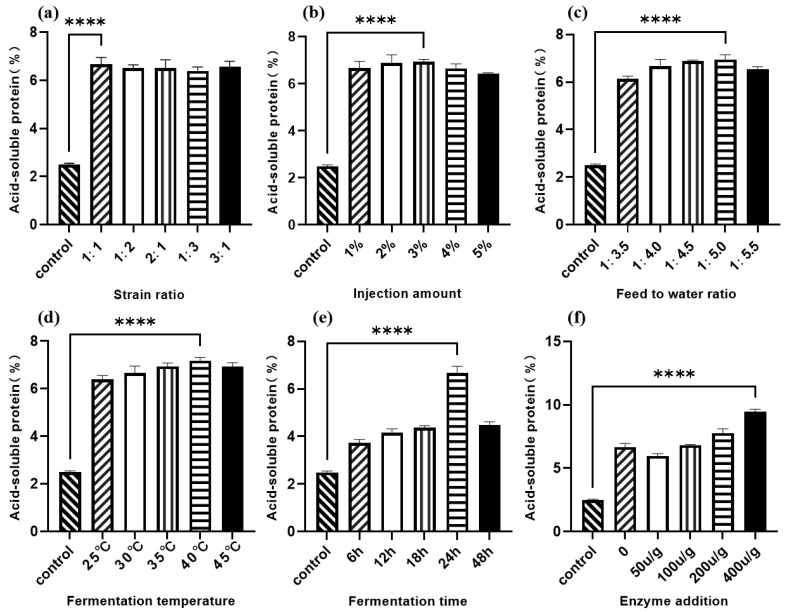
Single-factor optimization of sesame meal fermentation with acid-soluble protein content as the dependent variable. (**a**) Strain ratio; (**b**) injection amount; (**c**) feed to water ratio; (**d**) fermentation temperature; (**e**) fermentation time; (**f**) enzyme addition. **** *p* < 0.001.

As shown in Figure 3a, the acid-soluble protein content of *Lactobacillus* I and *C. utilis* at five levels after fermentation was significantly higher than that of the control group, and their values were basically the same. When the ratio was 1:1, the acid-soluble protein content was most significant compared to the control group. According to Figure 3b, as the inoculation amount increases, the content of acid-soluble protein in sesame meal after fermentation first increases and then decreases. At an inoculation amount of 3% (*v*/*w*), the content of acid-soluble protein reaches its maximum value. The trend of the effect of the feed water ratio on the acid-soluble protein content of sesame meal is roughly the same as that of the inoculation amount. As shown in Figure 3c, the change in acid-soluble protein content is most significant when the feed water ratio is 1:5; Figure 3d shows the effect of fermentation temperature on the acid-soluble protein content of sesame meal after fermentation, with 40 °C being the optimal fermentation temperature. According to Figure 3e, the change in acid-soluble protein content is most significant at 24 h, which is the optimal fermentation time for sesame meal. As shown in Figure 3f, the acid-soluble protein content is highest when the protease addition amount is 400 u/g.

#### 3.1.3. RSM Optimization of Fermentation Settings

Multiple regression analysis of the experimental data using Design-Expert software (Version 12.0.0) generated the following polynomial quadratic equation describing ASP content (Y) in fermented walnut meal as a function of the independent variables:

Y = 13.31 + 0.2458A + 0.2592B + 1.12C − 1.25D − 0.515AB − 1.1AC + 0.4875AD + 0.45BC + 0.9725BD − 0.1CD − 0.4192A^2^ − 0.2917B^2^ + 0.297C^2^ + 1.36D^2.^

In this equation, Y represents ASP content, while A, B, C, and D correspond to inoculum volume, water-to-material ratio, fermentation temperature, and fermentation time, respectively. ANOVA results for the response surface data (Table 2) revealed that the regression model was highly significant (*p* < 0.001) and exhibited a non-significant lack-of-fit term (*p* = 0.578). Among the model terms, C (fermentation temperature), D (fermentation time), AB (inoculum volume × water-to-material ratio interaction), AC (inoculum volume × temperature interaction), BD (water-to-material ratio × time interaction), A^2^ (quadratic inoculum volume), and D^2^ (quadratic fermentation time) significantly influenced ASP content (*p* < 0.05). The high coefficient of determination (R^2^ = 0.9556) and adjusted R^2^ (Adj. R^2^ = 0.9113) indicated excellent model fitting and strong agreement with experimental observations, supporting its suitability for theoretical predictions.

**Figure 4:**
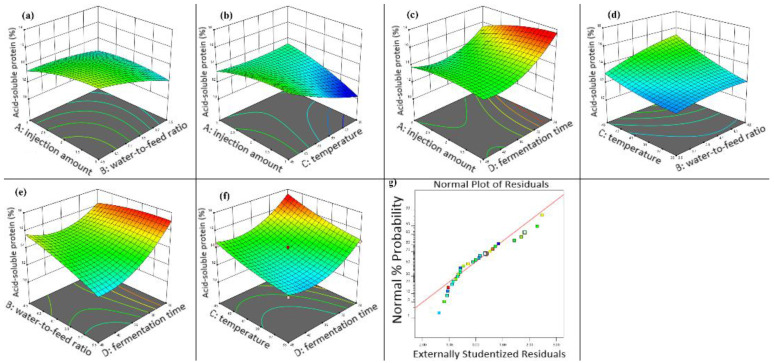
Three-dimensional response surface diagram of the effect of fermentation conditions on the hemicellulose content in walnut meal. (**a**) Interaction between injection amount and water to feed ratio; (**b**) interaction between injection amount and fermentation temperature; (**c**) interaction between injection amount and fermentation time; (**d**) interaction between fermentation temperature and water to feed ratio; (**e**) interaction between water to feed ratio and fermentation time; (**f**) interaction between fermentation temperature and fermentation time; (**g**) walnut meal fermentation residual fitting model diagram.

Figure 4a shows that, at fixed inoculum levels, higher water-to-material ratios increased hemicellulose content in fermented walnut meal. Similarly, Figure 4b demonstrates that hemicellulose content increased with increasing fermentation temperature at constant inoculum volume. Figure 4c illustrates that longer fermentation duration resulted in decreased hemicellulose content when inoculum volume was held constant, highlighting the interaction between fermentation time and inoculum volume. As shown in Figure 4d, maintaining a constant water-to-material ratio yielded higher hemicellulose content at elevated fermentation temperatures. Figure 4e,f depict the interactions of fermentation time with water-to-material ratio and fermentation temperature, respectively, revealing that hemicellulose content increased with both higher water-to-material ratios and elevated fermentation temperatures at fixed fermentation duration. The residual versus predicted plot (Figure 4g) indicated good model fit, with residuals primarily following a linear pattern with minor deviations clustered closely.

The final fermentation conditions were selected based on the statistical optimization from the RSM model, which identified the ideal fermentation parameters as: inoculum volume of 1.068% (*v*/*w*), water-to-material ratio of 4.057, fermentation temperature of 44.061 °C, and fermentation time of 24.028 h, predicting a maximum ASP content of 17.799%.

The polynomial quadratic equation with the acid-soluble protein content after sesame meal fermentation as the dependent variable:

Y = 10.31 − 0.0112A + 1.15B + 0.7412C + 0.39AB + 0.4725AC − 0.505BC − 0.5363A^2^ + 0.9512B^2^ + 0.4388C^2^

The Y represents the content of acid-soluble protein, and A, B, C, and D represent the inoculation amount, fermentation time, and fermentation temperature, respectively. The results of the analysis of variance on the response surface test data are shown in Table 3. The regression model shows significant differences (*p* < 0.01), and equations B, C, AB, AC, BC, A^2^, B^2^, and C^2^ have a significant impact on acid-soluble protein content (*p* < 0.05). The R^2^ of the model is 0.9836, indicating a good degree of fitting. The adjusted R^2^ is 0.9625, indicating that the regression equation fits well and is consistent with the actual situation. The model can be used for theoretical prediction of optimal fermentation conditions.

**Figure 5:**
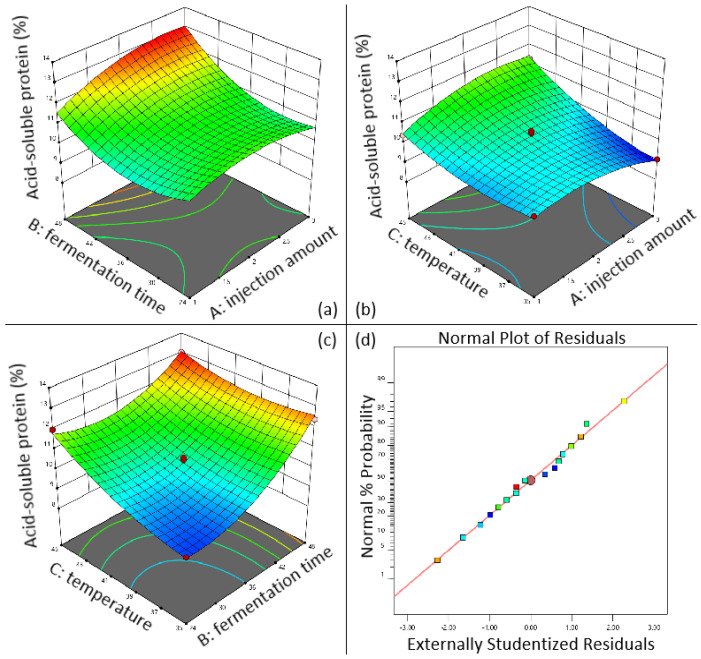
3D response surface diagram of the effect of fermentation conditions on acid-soluble protein content in sesame meal. (**a**) Interaction between injection amount and fermentation time; (**b**) interaction between injection amount and fermentation temperature; (**c**) interaction between fermentation temperature and fermentation time; (**d**) sesame meal fermentation residual fitting model diagram.

As shown in Figure 5a, when the inoculation amount is fixed, the longer the fermentation time, the higher the content of acid-soluble protein in sesame meal; Figure 5b shows the interactive effect of inoculation amount and fermentation temperature on the content of acid-soluble protein in sesame meal. When the inoculation amount is fixed, the higher the fermentation temperature, the higher the content of acid-soluble protein; Figure 5c shows the interaction between fermentation time and fermentation temperature. In the residual fitting model diagram (Figure 5d), most of the residual points are distributed on a straight line, indicating a high degree of model fitting and no significant mismatch term (*p* = 0.401). It can be seen that when the fermentation temperature is fixed, the longer the fermentation time, the higher the acid-soluble protein content. When the fermentation time is fixed, the higher the fermentation temperature, the higher the acid-soluble protein content. According to the results, the optimal fermentation conditions for sesame meal are as follows: inoculation volume of 2.935% (*v*/*w*), fermentation time of 47.997 h, fermentation temperature of 44.90 °C, and predicted acid-soluble protein content of up to 13.38%.

#### 3.1.4. Variations in Nutritional Quality After Fermentation

After fermentation, the crude protein content of walnut meal and sesame meal significantly increased, by 10.63% and 7%, respectively, 47% (*p* < 0.01), the tannin content in walnut meal decreased by 39.41%, and the phytic acid content in sesame meal decreased by 18.66% (*p* < 0.01). Concentrations of tryptophan, alanine, and cysteine were also significantly elevated in FWM (*p* < 0.05), while the level of isoleucine, leucine, valine, alanine, aspartic acid, glutamic acid, glycine, and proline in FSM shows an increasing trend (*p* < 0.10) (Table 4).

#### 3.1.5. Effects of Fermentation on the Surface Structure

Effects of fermentation on the surface structure are presented in Figure S1. It can be observed that the unfermented walnut meal has a smooth and compact structural surface, with a fibrous-like structure. After fermentation, the walnut meal appears in an irregular and loose state, and the fermented SM has a rougher surface and more pores compared to the unfermented SM. FTIR spectra displayed characteristic absorption bands near 2900 cm^−1^ (C-H stretching of polysaccharides), within the 1600–1700 cm^−1^ range (amide I band), and at 1066 cm^−1^ (C-O-C stretching of monosaccharides).

#### 3.1.6. Variations in Microbial Community

**Figure 6:**
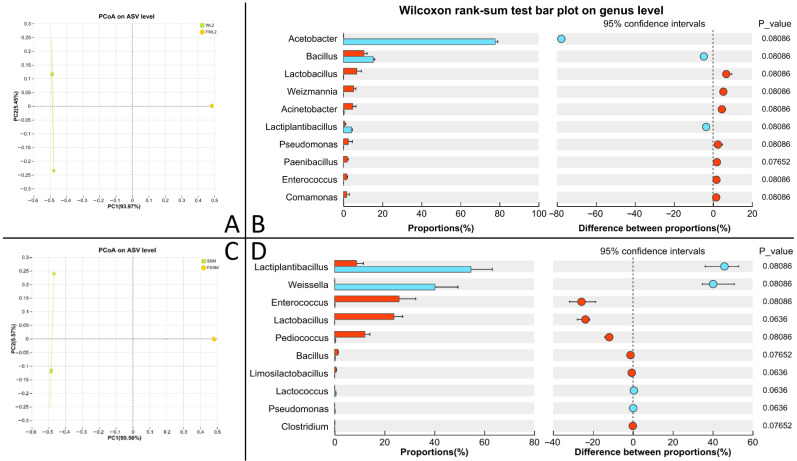
Results of microbial community of WM and SM before and after fermentation. (**A**) β diversity analysis of WM; (**B**) differential bacteria of WM; (**C**) β diversity analysis of SM; (**D**) differential bacteria of SM.

Alpha diversity of the microbial community is presented in Table S5. Fermented WM has higher Ace, Chao, and Sobs indices than unfermented WM, while fermented SM has lower Ace, Chao, and Sobs indices than unfermented SM (*p* < 0.05). Beta diversity analysis (Figure 6A,C) indicated distinct clustering between microbial communities in the raw and fermented substrates.

After the fermentation of WM, the relative abundance of *Lactiplantibacillus* and Weissella significantly decreased, while the relative abundance of *Enterococcus*, *Lactobacillus*, *Pediococcus*, *Bacillus*, and *Lactococcus* significantly increased (*p* < 0.05) (Figure 6B). After the fermentation of SM, the relative abundance of *Acetobacter*, *Bacillus*, and *Lactiplantibacillus* significantly decreased, while the relative abundance of *Lactobacillus*, *Weizmannia*, *Acinetobacter*, *Pseudomonas*, *Paenibacillus*, *Enterococcus*, and *Comamonas* significantly increased (*p* < 0.05) (Figure 6D).

### 3.2. Ileal Amino Acids Digestibility

Table 5 presents ileal AID and SID digestibility coefficients of amino acids in WM and SM, comparing values before and after fermentation in growing pigs. After fermentation, the AID of tryptophan and glycine in WM significantly increased (*p* < 0.05), while the SID of threonine, tryptophan, alanine, valine, glycine, and serine significantly increased (*p* < 0.05). The SID of arginine, isoleucine, leucine, and phenylalanine showed an increasing trend (*p* < 0.10).

After fermentation, the AID of isoleucine, leucine, valine, and alanine in SM significantly increased (*p* < 0.05), while the AID of lysine, phenylalanine, and tyrosine showed an increasing trend (*p* < 0.10). The SID of isoleucine, leucine, phenylalanine, valine, alanine, glycine, and tyrosine were significantly increased (*p* < 0.05), while the SID of lysine and threonine showed an increasing trend (*p* < 0.10).

### 3.3. Nutrient Digestibility, Digestible Energy, and Metabolic Energy

Table 6 presents the digestibility coefficients, DE, and ME values of WM and SM for growing pigs, comparing values before and after fermentation. The digestibility of CP, EE, and CF in the WM diet did not show significant changes (*p* > 0.10), while the digestibility of NDF and ADF significantly increased in the fermented WM diet (*p* < 0.05). The digestibility of CP, EE, and CF in the SM diet was significantly increased (*p* < 0.05), while the digestibility of NDF and ADF did not change significantly (*p* > 0.10). After fermentation, the DE and ME of the SM diet significantly increased (*p* < 0.05), while that of the WM diet did not change significantly (*p* = 0.40).

## 4. Discussion

Within the animal intestine, LAB are widely employed in the fermentation of feed materials and are recognized as a crucial beneficial bacterial group [27]. Their primary contribution to fermentation lies in their capacity to effectively regulate the process. Following rapid proliferation, LAB generate substantial quantities of lactic acid, thereby lowering the pH, suppressing the growth of undesirable bacteria, and enhancing the hygienic quality of liquid fermentation environments [28,29,30,31]. Prior research indicates that LAB possess a notable capability for phytic acid degradation. For instance, experimental findings by Lopez et al. (2000) demonstrated that LAB can decompose phytic acid salts, leading to an increase in inorganic phosphate content [32]. Further substantiating this, Lau et al. (2022) provided experimental evidence confirming LAB’s phytic acid-degrading ability [33]. Their study, involving anaerobic fermentation of liquid feed with three LAB types (*L. plantarum*, *S. pentosus*, and *L. lactis*), revealed a reduction in phytic acid phosphorus content from 3.07% to 2.66%. Moreover, LAB contribute to tannin degradation; specifically, *L. plantarum* has been shown to produce tannase [34]. Curiel et al. (2009) isolated and characterised tannase produced by *L. plantarum*, observing high enzymatic activity at 40 °C [35]. In a related study, Shang et al. (2019) found that fermenting papaya with *Lactobacillus* resulted in a 78% tannin removal rate [36]. The results from the present study indicated a 39.41% reduction in tannin content in walnut meal treated via liquid fermentation enzymatic hydrolysis compared to untreated walnut meal, and an 18.66% decrease in phytic acid content in treated sesame meal relative to its untreated counterpart. Aligning with established previous research, the findings of this study demonstrate that *Lactobacillus* spp. significantly contribute to the degradation of tannin and phytic acid, two prevalent anti-nutritional factors. This finding is likely due to the significant enrichment of *Enterococcus*, *Lactobacillus*, and *Bacillus* in the fermented WM microbial community. To elaborate, *Enterococcus* produces tannin-degrading enzymes, and *Lactobacillus* and *Bacillus* secrete cellulase and xylanase, thereby facilitating fibre degradation [28,29]. Conversely, the microbial profile of fermented SM was characterised by a dominance of *Lactobacillus*, *Weizmannia*, and *Acinetobacter*. Within this composition, *Lactobacillus* secretes phytase, and *Weizmannia* and *Acinetobacter* contribute to microbial protein synthesis and the generation of various organic acids [29,30,31].

Yeast is a widely recognized fermentation strain, particularly prevalent in the feed industries [37]. Cao et al. (2024) demonstrated that fermenting soybean meal with brewing strains of yeast resulted in enhanced crude protein and acid-soluble protein content, reaching 542.5 g/kg and 117.2 g/kg, respectively [38]. Further investigation by Anderson et al. (2015) into yeast protein synthesis from glucose revealed that supplementing with 25 mM inorganic phosphate yielded an almost threefold increase in protein production [39]. Moreover, studies using *C. utilis* and *B. subtilis* to ferment Huangjiu lees reported a 14.5% rise in crude protein. In this study, enzymatic fermentation led to a 10.63% increase in the crude protein content of walnut meal and a 7.47% increase in sesame meal [40]. These results are in concordance with previous research findings. However, the increase in crude protein content may be attributed to the fermentation process, in which microorganisms utilize a portion of the soluble carbohydrates in the feed as an energy source, decompose them to produce carbon dioxide and water that are released into the air, resulting in a reduction in the total dry matter of the feed and thus creating a concentration effect [11,12]. Alternatively, during their growth and reproduction, the fermenting strains may utilize non-protein nitrogen, small-molecule peptides, and amino acids from the feed to synthesize their own microbial biomass protein [20,22]. When the amount of protein synthesized by the microorganisms exceeds that decomposed and consumed from the raw materials, the total protein content in the feed would increase accordingly [39,40]. Therefore, it is essential to combine the nutrient digestibility data to evaluate the actual effect of nutritional improvement.

Variance analysis of the response surface optimization experiment for walnut meal indicated that fermentation temperature had the most significant influence on acid-soluble protein content. This may be attributed to walnut meal’s composition, which is rich in cellulose and tannins, resulting in a compact fibrous structure that inherently limits microbial enzyme accessibility to the substrate. As temperature serves as the principal factor governing enzymatic activity, it consequently emerged as the most impactful variable in enhancing protein solubility. Following temperature, fermentation time, water-to-material ratio, and inoculum size were identified as successively less influential factors in the optimization model. For sesame meal fermentation, the primary anti-nutritional factor in sesame meal is phytic acid, and microbial protein synthesis is more dependent on the duration of microbial metabolism, which renders fermentation time the most influential factor on acid-soluble protein content. Following fermentation time, temperature and bacterial enzyme inoculation amount were identified as successively less influential factors in the optimization model. A principal advantage of liquid fermentation over solid fermentation is its reduced fermentation period [27]. Prior research has demonstrated that the accumulation of lactic acid by lactic acid bacteria during liquid fermentation shows a significant difference compared to the control group after 8 h. By 16 h, lactic acid accumulation approximates its maximum value and stabilizes [33].

Surface structures of both feed materials were observed to have changed to varying degrees via scanning electron microscopy. The microstructural alterations (surface roughness, porosity) and chemical bond modifications (e.g., in polysaccharide and protein regions) revealed by SEM and FTIR analyses are closely linked to potential improvements in nutrient digestibility. These physical and chemical structural changes are hypothesized to increase the accessibility of digestive enzymes to protein and carbohydrate substrates, thereby enhancing nutrient release and absorption. Untreated walnut meal presented with a compact structure and smooth surface, whereas the fermented feed materials displayed a rougher surface and reduced particle size, potentially due to fermentation strain metabolic products or the inclusion of proteases [41,42]. For the result of FTIR spectra, both feed ingredients exhibited significantly enhanced absorption peaks at 2930 cm^−1^, indicative of the stretching vibration of C-H bonds. This observed peak enhancement is likely attributable to the degradation of polysaccharides during fermentation, which would expose more CH_2_ and CH_3_ groups due to the breakage of long chains [43]. Following fermentation, both walnut and sesame meals displayed more pronounced absorption peaks around 1060 cm^−1^, a region associated with the C-O-C ether bond stretching vibration within sugar units [44]. Based on single-factor fermentation outcomes, this spectral change may result from the metabolic activity of the fermentation strain or the breakdown of hemicellulose by cellulase, leading to the depolymerisation of polysaccharides into smaller oligosaccharides or structural modifications [45]. Furthermore, the observed FTIR spectral changes in fermented WM may be attributed to the enrichment of *Bacillus* and *Lactobacillus*. These microorganisms secrete cellulase and xylanase, which cleave long-chain fibres, induce polysaccharide depolymerisation, expose additional CH_2_/CH_3_ groups, and thereby directly enhance the two characteristic peaks. Conversely, the dominant *Lactobacillus* in fermented SM exhibits weak enzyme-producing capacity, resulting in limited hemicellulose decomposition and only a mild enhancement of the FTIR characteristic peaks.

Microbial community shifts in walnut meal before and after fermentation were evaluated using alpha and beta diversity indices and differential abundance profiling. The integrated analysis of alpha and beta diversity demonstrated that fermentation induced substantial alterations in microbial composition, leading to a notable increase in microbial abundance within the fermented substrate. This enrichment is likely attributed to the initial low moisture content of unfermented WM and SM, which inherently restricted robust microbial proliferation [41]. Post-fermentation, the microbial community displayed enhanced specialization, with dominant taxa stabilizing within the *Lactobacillus* and *Bacillus* genera. Notably, *Weissella* species metabolize glucose for lactic acid production and thrive optimally at pH values ≤ 5.5 [46], while *L. plantarum* modifies carbohydrate metabolism to boost ATP generation and can further adjust amino acid metabolism under acidic conditions [47,48,49]

Following fermentation, the ileal digestibility of amino acids in walnut and sesame meals exhibited an upward trend. This improvement may be attributed to the elevated levels of acid-soluble protein, specifically free peptides and amino acids, post-fermentation [50]. Existing research indicates that an increase in acid-soluble protein content positively influences the digestibility of crude protein and amino acids [51], which was consistent with a current study where probiotics metabolize and utilize proteins in the feed to produce acid-soluble proteins, which improving palatability, facilitating the digestion or absorption of piglets, thus ensuring the utilization and quality of fermented feed [52]. Previous studies reported apparent ileal digestibility values for lysine, methionine, and threonine in feed supplemented with casein-sesame meal as 85.3%, 85.4%, and 85.3%, respectively [52]. While the apparent ileal digestibility of methionine in this experiment was comparable, the values for lysine and threonine were lower. This discrepancy might be attributable to an imbalance in the amino acid profile of sesame meal when used as a sole dietary component [50,52]. Notably, the observation that the SID of glycine in FWM surpassed 100% (103.82%) is a significant and not uncommon finding in studies of fermented ingredients. This phenomenon strongly suggests the net entry of glycine into the digestive stream beyond what was intrinsically present in the FWM itself. The most scientifically supported explanation for this is likely the synthesis of glycine by the gut microbiota in the ileum, followed by its absorption. Functional shift in the gut environment induced by fermentation. The altered microbial community likely facilitates the in situ production and subsequent absorption of glycine [49]. This underscores a key benefit of fermented ingredients: their impact extends beyond simply improving the digestibility of intrinsic nutrients to actively enriching the nutrient pool within the gut through microbial metabolism [47,48].

The crude protein digestibility of walnut meal demonstrated an upward trend following fermentation treatment. This improvement is attributable not only to the increased acid-soluble protein content but also, in part, to the degradation of tannin [53,54]. Research indicates that tannins impair animal nutrient digestion and absorption by hydrolyzing gastric and pancreatic proteases and inhibiting ileal microbial degradation [55]. Comparative study from Pan et al. (2022) on pigs fed sorghum with high versus low tannin content revealed significantly lower nutrient digestibility in the high-tannin group [56], a finding that aligns with the results of this study. Furthermore, the digestibility of nutrients such as crude protein and crude fibre in fermented sesame meal was significantly higher than in untreated samples, with the degradation of the anti-nutritional factor phytic acid playing a crucial role [57]. Phytic acid’s potent chelating effect with metal ions forms phytate salts, which inhibit gastric proteases and consequently hinder mineral and protein digestion and absorption in animals [58,59]. Lagos et al. (2022) demonstrated that phytase supplementation effectively degrades phytate content, leading to a significant enhancement in amino acid and crude protein digestibility in pigs [60]. Similarly, this experiment observed an increasing trend in the digestion rate of crude ash in fermented sesame meal, potentially owing to phytic acid degradation, which consequently improved mineral digestion and utilization by pigs [61]. Research supporting this, which suggests reduced phytate content benefits calcium and phosphorus absorption and lowers phosphorus excretion in pigs [62], is consistent with these results.

The digestive and metabolic energy of sesame meal underwent significant changes after fermentation treatment, whereas walnut meal displayed an increasing trend in these energy metrics. Existing research suggests that digestive energy metabolism is negatively correlated with neutral and acid detergent fibre content and positively correlated with crude protein levels [63]. Moreover, a predictive equation developed for sorghum’s digestive and metabolic energy demonstrated that tannin content also influences these values [64]. This association may be due to the considerable effect of tannin on protein digestibility, which, in turn, indirectly impacts the feed’s digestive and metabolic energy.

This study has several limitations. The sample size, ten castrated male crossbred pigs used across both experiments, may limit statistical power and the ability to detect smaller treatment effects. Furthermore, the research did not assess key practical aspects such as animal growth performance, intestinal health, economic feasibility, or the scalability of the fermentation process. Future studies should include large-scale feeding trials to evaluate growth metrics (e.g., ADG, FCR), intestinal morphology and microbiota, and the consistency, cost, and scalability of production. Such work would strengthen the practical applicability of fermented wheat middlings and soybean meal in swine diets.

## 5. Conclusions

A novel liquid-state fermentation process for walnut and sesame meals was developed and optimized via single-factor and response surface methodology experiments. This process utilizes a synergistic microbial-enzymatic starter culture composed of a multi-strain cocktail (*Lactobacillus*, *C. utilis*, and protease). In-depth analyses of proximate composition, amino acid profiles, microbiota, ultrastructure, and protein conformation demonstrated that this consortium-based liquid fermentation significantly reduced anti-nutritional tannin and crude fibre, while improving overall nutritional quality. Further evaluation in growing pigs, using SID, AID, DE, and ME values, validated the enhanced nutritional value of the fermented walnut meal. Consequently, these results support the utilization of liquid-state, microbial-enzymatically fermented walnut and sesame meals as beneficial swine feed ingredients. Furthermore, long-term feeding trials encompassing different pig breeds and distinct growth stages (e.g., weaner, grower, finisher) are warranted to thoroughly evaluate the impacts on overall growth performance, carcass traits, and meat quality. Such investigations would provide critical data for optimizing dietary formulations and offer novel insights for the development of high-quality, value-added compound feeds.

## Figures and Tables

**Table 1:** Polynomial regression equation between bacterial count and OD600 value.

Strain	Regression Equation	R^2^
*Lactobacillus* (×108)	Y = 2.1731X^2^ + 3.9172X − 4.9534	0.9975
*Lactobacillus* II (×107)	Y = 40.405X^2^ − 54.217X + 24.425	0.9908
*S. cerevisiae* (×106)	Y = 9.0768X^2^ + 12.622 + 1.4077	0.9979
*C. utilis* (×106)	Y = −234.53X^2^ + 636.31X − 379.53	0.9891

**Table 2:** Variance analysis of fitted model for acid-soluble protein content in walnut meal after fermentation.

Source	Sum of Squares	Degree of Freedom	Mean Square	F-Value	*p*-Value
Model	63.63	14	4.55	21.54	<0.0001
A-injection amount	0.7252	1	0.7252	3.44	0.0849
B-water to feed ratio	0.8060	1	0.8060	3.82	0.0709
C-fermentation temperature	15.05	1	15.05	71.34	<0.0001
D-fermentation time	18.90	1	18.90	89.57	<0.0001
AB	1.06	1	1.06	5.03	0.0417
AC	4.84	1	4.84	22.94	0.0003
AD	0.9506	1	0.9506	4.51	0.0521
BC	0.8100	1	0.8100	3.84	0.0703
BD	3.78	1	3.78	17.93	0.0008
CD	0.0400	1	0.0400	0.1896	0.6699
A^2^	1.14	1	1.14	5.40	0.0357
B^2^	0.5521	1	0.5521	2.62	0.1280
C^2^	0.5722	1	0.5722	2.71	0.1219
D^2^	11.94	1	11.94	56.61	<0.0001
Residual	2.95	14	0.2110		
Lack of Fit	2.07	10	0.2070	0.9368	0.5781
Pure Error	0.8839	4	0.2210		
Cor Total	66.59	28			
R^2^	0.9556				
Adj. R^2^	0.9113				

**Table 3:** Variance analysis of fitted model for acid-soluble protein content in sesame meal after fermentation.

Source	Sum of Squares	Degree of Freedom	Mean Square	F-Value	*p*-Value
Model	23.16	9	2.57	46.68	<0.0001
A-injection amount	0.0010	1	0.0010	0.0184	0.8960
B-water to feed ratio	10.53	1	10.53	191.04	<0.0001
C-fermentation temperature	4.40	1	4.40	79.72	<0.0001
AB	0.6084	1	0.6084	11.03	0.0127
AC	0.8930	1	0.8930	16.20	0.0050
BC	1.02	1	1.02	18.50	0.0036
A^2^	1.21	1	1.21	21.96	0.0022
B^2^	3.81	1	3.81	69.10	<0.0001
C^2^	0.8105	1	0.8105	14.70	0.0064
Residual	0.3860	7	0.0551		
Lack of Fit	0.1872	3	0.0624	1.26	0.4014
Pure Error	0.1988	4	0.0497		
Cor Total	23.55	16			
R^2^	0.9836				
Adj. R^2^	0.9625				

**Table 4:** Comparison of nutrient content of WM and SM before and after fermentation (DM%).

Item	WM	FWM	SEM	*p*	SM	FSM	SEM	*p*
CP	40.53	44.84	0.48	<0.01	37.06	39.83	0.25	<0.01
EE	17.69	19.87	0.79	0.05	17.69	17.97	0.41	0.54
CF	10.70	7.62	0.36	<0.01	10.26	9.85	0.54	0.50
NDF	17.43	14.82	0.24	<0.01	30.51	22.80	0.76	<0.01
ADF	11.71	10.04	0.20	<0.01	16.89	15.16	0.35	<0.01
Ash	6.34	7.57	0.08	<0.01	14.48	14.71	0.60	0.73
Ca	1.52	1.47	0.22	0.83	1.89	2.17	0.05	<0.01
P	1.06	1.05	0.02	0.62	1.00	0.99	0.03	0.66
Tannin	17.46	10.58	0.67	<0.01	6.59	5.36	0.14	<0.01
Arginine	4.58	4.39	0.20	0.39	2.70	3.35	0.52	0.28
Histidine	0.89	0.88	0.04	0.68	0.56	0.77	0.11	0.12
Isoleucine	1.27	1.36	0.05	0.15	0.85	1.24	0.16	0.08
Leucine	2.56	2.67	0.11	0.38	1.7	2.41	0.32	0.09
Lysine	1.40	1.25	0.06	0.06	0.43	0.55	0.08	0.23
Methionine	0.39	0.46	0.03	0.08	0.59	0.85	0.12	0.11
Phenylalanine	1.53	1.55	0.07	0.82	1.15	1.58	0.21	0.12
Threonine	1.33	1.31	0.06	0.82	0.86	1.20	0.17	0.12
Tryptophan	0.45	0.82	0.01	<0.01	0.93	0.87	0.03	0.12
Valine	1.55	1.66	0.07	0.17	1.12	1.63	0.22	0.08
Alanine	1.56	1.79	0.07	0.03	1.29	1.90	0.25	0.07
Aspartate	3.28	3.52	0.14	0.17	1.95	2.84	0.38	0.08
Cystine	0.43	0.62	0.02	<0.01	-	0.17	-	-
Glutamate	7.76	8.26	0.34	0.21	5.36	7.63	1.03	0.09
Glycine	1.86	2.01	0.08	0.14	1.28	1.88	0.25	0.07
Proline	5.91	6.32	0.27	0.21	4.17	5.87	0.78	0.09
Serine	1.88	1.86	0.08	0.85	1.01	1.38	0.20	0.15
Tyrosine	1.20	1.18	0.05	0.70	1.01	1.31	0.17	0.16
Total AA	39.84	41.91	1.72	0.29	26.98	37.44	4.99	0.10

**Table 5:** Ileal amino acid digestibility before and after fermentation of WM and SM (DM %).

Item	WM	FWM	SEM	*p*	SM	FSM	SEM	*p*
**AID**								
Crude Protein	72.03	72.07	3.85	0.99	67.15	67.70	6.68	0.94
Arginine	96.61	97.11	0.56	0.39	87.36	87.74	1.44	0.79
Histidine	92.75	94.16	1.19	0.26	76.60	80.03	3.04	0.29
Isoleucine	93.99	95.16	0.75	0.15	74.07	81.13	2.72	0.04
Leucine	94.11	95.19	0.77	0.19	75.92	82.20	2.39	0.04
Lysine	90.95	91.66	1.39	0.62	40.13	52.77	5.84	0.07
Methionine	95.64	95.67	0.84	0.98	83.41	85.20	2.13	0.43
Phenylalanine	95.23	95.95	0.57	0.24	78.64	82.26	1.78	0.07
Threonine	91.76	93.12	0.98	0.19	64.56	70.76	4.50	0.20
Tryptophan	89.07	95.95	1.97	<0.01	73.04	78.22	5.22	0.34
Valine	93.11	94.51	0.84	0.13	72.71	80.88	3.01	0.04
Alanine	92.89	94.19	0.77	0.12	66.35	77.76	3.04	0.01
Aspartate	95.07	95.83	0.70	0.30	64.22	68.79	5.95	0.46
Cystine	89.87	90.85	1.76	0.59	45.01	55.81	8.93	0.25
Glutamate	95.25	95.70	0.94	0.64	79.67	81.32	4.02	0.69
Glycine	87.98	92.40	1.56	0.02	66.65	72.93	4.44	0.19
Proline	95.22	95.66	1.02	0.67	79.41	82.21	4.49	0.55
Serine	93.65	94.78	0.88	0.23	73.54	75.58	3.62	0.59
Tyrosine	94.15	94.79	0.83	0.46	78.77	82.29	1.65	0.06
**SID**								
Crude Protein	76.47	76.34	3.85	0.97	73.04	73.59	6.68	0.94
Arginine	97.56	98.64	0.56	0.08	88.89	90.54	1.44	0.28
Histidine	94.61	96.54	1.19	0.13	79.33	83.86	3.04	0.17
Isoleucine	95.88	97.48	0.75	0.06	76.49	84.24	2.72	0.03
Leucine	95.82	97.35	0.77	0.07	78.16	85.21	2.39	0.03
Lysine	93.54	94.88	1.39	0.36	46.31	60.58	5.84	0.05
Methionine	96.84	96.99	0.84	0.87	84.16	86.15	2.13	0.38
Phenylalanine	96.88	97.94	0.57	0.09	80.51	84.72	1.78	0.04
Threonine	94.92	97.93	0.98	0.01	68.69	77.77	4.50	0.07
Tryptophan	93.04	99.58	1.97	0.01	76.58	82.50	5.22	0.28
Valine	95.37	97.43	0.84	0.03	75.40	84.50	3.01	0.02
Alanine	96.08	98.61	0.77	0.01	69.83	82.77	3.04	0.01
Aspartate	96.93	98.18	0.70	0.10	67.11	72.68	5.95	0.37
Cystine	92.08	93.72	1.76	0.38	52.43	64.71	8.93	0.20
Glutamate	96.38	97.24	0.94	0.38	81.13	83.32	4.02	0.60
Glycine	94.88	103.82	1.56	<0.01	75.55	88.38	4.44	0.02
Proline	96.36	97.13	1.02	0.46	80.86	84.01	4.49	0.50
Serine	95.95	98.31	0.88	0.02	77.33	82.17	3.62	0.21
Tyrosine	96.07	97.33	0.83	0.16	80.83	85.19	1.65	0.02

**Table 6:** Ileal amino acid digestibility before and after fermentation of WM and SM (DM %).

Item	WM	FWM	SEM	*p*	SM	FSM	SEM	*p*
**Diet**								
DM	83.46	82.56	1.03	0.40	75.33	80.25	1.26	<0.01
CP	80.63	82.50	1.28	0.17	65.03	75.43	2.49	0.01
EE	91.29	90.38	1.17	0.45	84.87	89.29	1.30	0.01
CF	36.79	41.57	4.60	0.32	22.95	40.07	3.96	<0.01
NDF	52.89	67.14	3.22	<0.01	54.52	57.69	2.93	0.31
ADF	23.89	34.68	3.90	0.02	33.03	31.56	2.47	0.57
Ash	48.71	50.77	3.41	0.56	32.11	41.53	4.23	0.05
**Ingredient**								
DM	92.38	93.76	0.52	0.03	92.49	93.73	0.59	0.06
CP	90.95	92.27	0.98	0.21	90.53	92.10	1.02	0.15
EE	94.81	95.67	0.51	0.12	94.63	95.66	0.52	0.08
CF	75.42	77.90	3.08	0.44	75.16	78.00	3.06	0.38
NDF	84.20	85.76	1.25	0.24	83.90	86.03	1.29	0.13
ADF	64.47	69.55	3.10	0.13	65.60	69.96	3.32	0.22
Ash	77.11	79.12	1.28	0.15	76.73	78.96	1.40	0.14

## Data Availability

The data supporting the findings of this study are included within the article. Further inquiries can be directed to the corresponding author.

## Supplementary Materials

The following supporting information can be downloaded at: https://www.mdpi.com/article/10.3390/ani16020220/s1, Table S1: Experimental design for optimizing raw material fermentation conditions; Table S2: Experimental design for RSM; Table S3: Formula and nutrient content of diets in the trial of amino acids digestibility; Table S4: Dietary formula of the trial of nutrient digestibility. Table S5: Comparison of nutrient content of WM and SM before and after fermentation (DM%). Figure S1: Effects of fermentation on the surface structure. (a) Unfermented WM; (b) fermented WM; (c) unfermented SM; (d) fermented SM (1000x); (e) FTIR spectra.

## Abbreviations

The following abbreviations are used in this manuscript:

AA	Amino acid
ADF	Acid detergent fibre
ADG	Average daily gain
AID	Apparent ileal digestibility
ANOVA	Analysis of variance
ASP	Acid-soluble protein
BIAA	Basal endogenous ileal amino acid
CF	Crude fibre
CP	Crude protein
DE	Digestible energy
DM	Dry matter
EE	Ether Extract
FTIR	Fourier transform infrared Spectrophotometer
LAB	Lactic acid bacteria
LFF	Liquid-state fermentation
ME	Metabolizable energy
MRS	de Man, Rogosa and Sharpe Medium
NDF	Neutral detergent fibre
RSM	Response surface methodology
SID	Standardized ileal digestibility
SEM	Standard error of the mean
SM	Sesame meal
WM	Walnut meal
YPD	Yeast extract peptone dextrose medium
