# Supplementary material for: Liquid Microbial-Enzymatic Co-Fermentation of Walnut and Sesame Meals and Its Effects on Nutrient Digestibility in Growing Pigs
Source: Animals (Basel). 2026 Jan 12;16(2):220. doi: 10.3390/ani16020220 (PMC12838052; doi:10.3390/ani16020220)
Supplement: Supplementary file 1 [file animals-16-00220-s001.zip › animals-4062251-supplementary.pdf]

Supplementary Materials

Table S1. Experimental design for optimizing raw material fermentation conditions.

Factors	Level				
	1	2	3	4	5
Strain ratio	1:1	1:2	2:1	1:3	3:1
Injection amount	1%	2%	3%	4%	5%
Feed to water ratio	1:3.5	1:4.0	1:4.5	1:5.0	1:5.5
Fermentation temperature	25 °C	30 °C	35 °C	40 °C	45 °C
Fermentation time	6h	12h	18h	24h	48h
Enzyme addition	0u/g	50u/g	100u/g	200u/g	400u/g

Table S2. Experimental design for RSM.

Run order	Independent variables			
	Injection amount %	Water to feed ratio	Temperature °C	Time h
1	2(0)	4(0)	40(0)	36(0)
2	1(-1)	4(0)	45(1)	36(0)
3	1(-1)	4.5(1)	40(0)	36(0)
4	3(1)	4(0)	40(0)	48(1)
5	1(-1)	3.5(-1)	40(0)	36(0)
6	2(0)	4(0)	35(-1)	24(-1)
7	1(-1)	4(0)	35(-1)	36(0)
8	2(0)	4(0)	40(0)	36(0)
9	2(0)	4.5(1)	35(-1)	36(0)
10	2(0)	4.5(1)	40(0)	24(-1)
11	1(-1)	4(0)	40(0)	48(1)
12	2(0)	4(0)	40(0)	36(0)
13	2(0)	4(0)	45(1)	24(-1)
14	2(0)	3.5(-1)	45(1)	36(0)
15	1(-1)	4(0)	40(0)	24(-1)
16	3(1)	4(0)	45(1)	36(0)
17	2(0)	4.5(1)	40(0)	48(1)
18	3(1)	4(0)	35(-1)	36(0)
19	3(1)	4(0)	40(0)	24(-1)
20	3(1)	4.5(1)	40(0)	36(0)
21	3(1)	3.5(-1)	40(0)	36(0)
22	2(0)	3.5(-1)	35(-1)	36(0)
23	2(0)	4(0)	40(0)	36(0)
24	2(0)	3.5(-1)	40(0)	24(-1)
25	2(0)	4(0)	35(-1)	48(1)
26	2(0)	3.5(-1)	40(0)	48(1)
27	2(0)	4(0)	45(1)	48(1)
28	2(0)	4.5(1)	45(1)	36(0)
29	2(0)	4(0)	40(0)	36(0)

Table S3. Formula and nutrient content of diets in the trial of amino acids digestibility

Item	Control	WM	FWM	SM	FSM
Ingredient					
Corn starch	78.90	42.90	42.90	42.90	42.90
WM	-	40.50	-	-	-
FWM	-	-	40.50	-	-
SM	-	-	-	40.50	-
FSM	-	-	-	-	40.50
Sucrose	10.00	10.00	10.00	10.00	10.00
Soya-bean oil	3.00	3.00	3.00	3.00	3.00
CMC	4.00	-	-	-	-
Calcium carbonate	0.50	0.50	0.50	0.50	0.50
Dicalcium phosphate	2.20	2.20	2.20	2.20	2.20
Cr2O3	0.30	0.30	0.30	0.30	0.30
NaCl	0.40	0.40	0.40	0.40	0.40
Vitamin premix1	0.05	0.05	0.05	0.05	0.05
Mineral premix2	0.15	0.15	0.15	0.15	0.15
K2CO3	0.40	-	-	-	-
MgO	0.10	-	-	-	-
Total	100.00	100.00	100.00	100.00	100.00
Nutrient Content					
Crude Protein	0.28	20.05	20.82	15.09	15.10
Ca	0.53	0.75	0.80	0.96	1.54
Total phosphorus	0.45	0.72	0.75	0.69	0.67
Lysine	0.01	0.66	0.58	0.28	0.24
Methionine	0.00	0.13	0.10	0.20	0.14

Table S4. Dietary formula of the trial of nutrient digestibility

Ingredient	Control	WD	FWD	SM	FSM
Corn	68.52	66.40	66.40	63.02	63.02
Soybean meal	27.88	-	-	-	-
WM	-	30.00	-	-	-
FWM	-	-	30.00	-	-
SM	-	-	-	33.38	-
FSM	-	-	-	-	33.38
Lysine	0.45	0.45	0.45	0.45	0.45
Methionine	0.10	0.10	0.10	0.10	0.10
Threonine	0.12	0.12	0.12	0.12	0.12
Tryptophan	0.05	0.05	0.05	0.05	0.05
Choline chloride	0.15	0.15	0.15	0.15	0.15
CaCO3	0.45	0.45	0.45	0.45	0.45
CaHPO4	1.15	1.15	1.15	1.15	1.15
Vitamin premix1	0.03	0.03	0.03	0.03	0.03

NaCl	0.30	0.30	0.30	0.30	0.30
Cr2O3	0.30	0.30	0.30	0.30	0.30
Mineral premix	0.50	0.50	0.50	0.50	0.50
Total	100.00	100.00	100.00	100.00	100.00

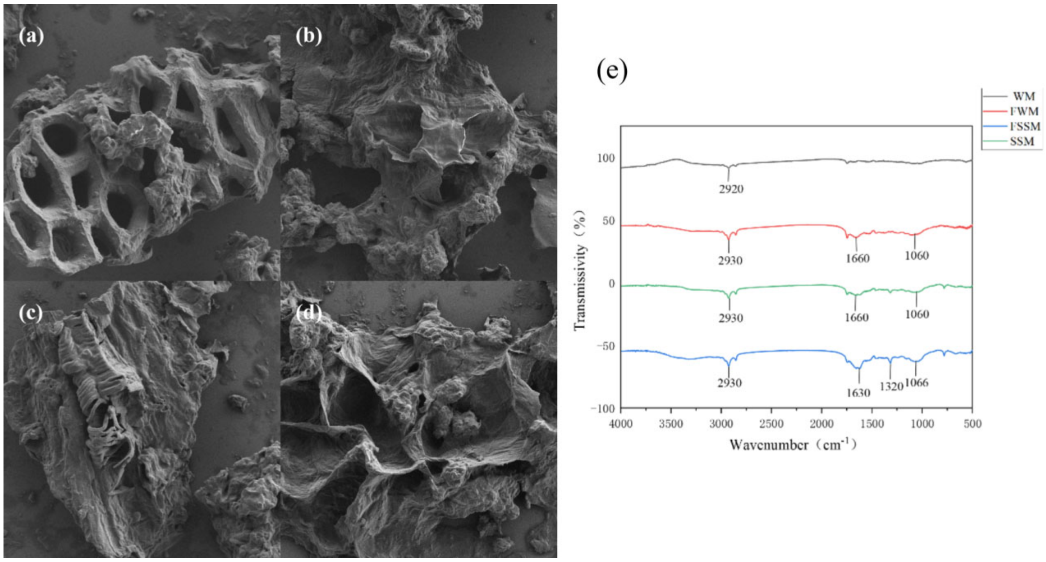

Figure S1. Effects of fermentation on the surface structure. (a) Unfermented WM; (b) fermented WM; (c) unfermented SM; (d) fermented SM (1000x); (e) FTIR spectra.

Table S5. Comparison of nutrient content of WM and SM before and after fermentation (DM%).

Item	WM	FWM	SEM	P	SM	FSM	SEM	P
Ace	45.39	69.45	7.69	0.04	817.49	115.87	18.19	< 0.01
Chao	44.21	68.15	6.71	0.02	809.14	111.74	15.45	< 0.01
Shannon	1.02	2.25	0.05	< 0.01	5.23	1.29	0.05	< 0.01
Simpson	0.46	0.15	0.02	< 0.01	0.01	0.54	0.01	< 0.01
Coverage	1	1	0	0.56	0.99	1	0	< 0.01
Sobs	43	65.33	5.21	0.01	743.67	104	12.41	< 0.01
